# CD47 on artificial structures

**DOI:** 10.18632/aging.100787

**Published:** 2015-08-15

**Authors:** Christian Schütz

**Affiliations:** Division of Immunology, Paul-Ehrlich-Institut, Langen, 63225, Germany

CD47, a “marker of self”, also referred to as integrin-associated protein (IAP) due to association with integrins such as α_V_β_3,_ has been first described by Brown et al. in 1990. As member of the immunoglobulin superfamily it contains one IgV-like domain and five transmembrane regions. While it is expressed on virtually all cell types, levels of expression are dependent on the immune status of cells. On leukemic cells for instance overexpression of CD47 has been discussed as tumour escape mechanism and thus intensively studied as tumour immunotherapy target. CD47 is involved in a variety of different cellular processes such as apoptosis, proliferation, adhesion and migration by interaction with thrombosponin-1 (TSP-1). However, binding properties to Signal regulating protein alpha (SIRPα), mainly expressed on macrophages and myeloid cells, made CD47 known under the designation of a “don't eat me” signal. Ligation of CD47 to SIRPα promotes the phosphory-lation of SIRPα Immunoreceptor Tyrosine-Based Inhibitory Motifs (ITIM) consequently leading to recruitment of the cytosolic tyrosine phosphatases SHP1 or SHP2. Activation of these phosphatases results in dephosphorylation of the motor protein myosin-II at the phagocytic synapse in macrophages, suppressing phagocytic processes [1, 2]. This CD47 characteristic is now in focus for drug delivery and nano-medicine research that is in need of strategies developing ‘stealth’ or inert artificial structures to avoid uptake by phagocytes and clearance.

Hsu et al. first investigated the potential of soluble CD47 to inhibit uptake of perfluorocarbon emulsions (PFC) by macrophages [3]. Streptavidin-CD47 fusion protein (CD47-SA) expressed in bacteria and purified by biotinylated agarose column, could prevent engulfment of PFC for up to 2 hours when macrophages (J774A.1) where incubated with CD47-SA for one hour prior to co-culture. Thus, this initial study showed sufficient inhibition of PFC phagocytosis by soluble CD47-SA but the authors just speculated that immobilization of CD47-SA onto the surface of PFC might even prolong the biological activity of CD47. This was tested in part by Tsai and colleagues which transfected Chinese hamster ovary cells with a extracellular domain of CD47 encoding plasmid [2]. The purified CD47 was biotinylated and coated onto streptavidin μm sized polystyrene beads. These beads were pseudo-opsonised by binding of anti-streptavidin IgG1 and tested for phagocytosis by THP-1 cells. Significant resistance to phagocytosis, mediated by myosin-II deactivation, could be demonstrated at a density of CD47 comparable to that on red blood cells (RBC). Rodriguez et al. could demonstrate inhibition of phagocytosis and delivery of nanoparticles by functionalization with a 21-amino acid ‘self’ peptide [4]. This peptide was generated from the 117-amino acid extracellular domain of CD47 by computer simulation and structural analysis. Immobilization of this ‘self’ peptide on 160 nm fluorescent polystyrene beads, resulted in a four times enhanced blood circulation time and *in vivo* delivery of paclitaxel to tumours by those beads demonstrated comparable tumour size reduction as by the standard paclitaxel carrier (i.e., Cremophor®). Most interestingly adverse toxic effects were significantly reduced at the same time. Recently, Bruns et al. functionalized bead-based, μm sized artificial antigen-presenting cells (aAPC) with an additional CD47-Ig fusion protein [5]. Those aAPC^CD47+^ showed reduced phagocytic uptake by human primary macrophages not interfering with antigen-specific T cell generation and expansion *in vitro*. At the same time *in vivo* studies demonstrated enhanced stimulatory capacity and tumour inhibition of aAPC^CD47+^ compared to aAPC, not functionalized with CD47-Ig. Together, these studies show that beads functionalized with CD47 or CD47-derived ‘self’ peptide inhibit phagocytic uptake by macrophages, which could translate into enhanced functionality.

Another approach to functionalize nanoparticles with CD47 was described by Hu et al [6]. 70 nm poly(lactic-*co*-glycolic acid) (PLGA) particles were cloaked with RBC derived membranes displaying the natural orchestra of immune modulatory proteins including CD47. Those particles showed reduced phagocytic uptake when co-cultured with macrophages (J774). This effect could be partially blocked by anti-CD47 revealing that the particle's ‘stealth’-properties were at least in part mediated by RBC membrane embedded CD47. Additionally, Stachelek et al. could demonstrate that implant surfaces such as polyvinyl chloride (PVC) and polyurethane (PU) coated with CD47 showed significantly reduced attachment of human neutrophils and macrophages (THP-1) [7]. In tests under clinical relevant conditions with CD47-coated PVC blood conduits, Finley et al. demonstrated significantly reduced attachment and activation of platelets and neutrophils by those artificial implant structures compared to non-functionalized structures [8]. Therefore, CD47-functionalized artificial surfaces provide a superior opportunity for clinical application minimizing inflammatory cell interaction such as adhesion and activation.

CD47 functionalization has become a very promising approach to develop long-circulating, immune-evasive artificial structures. The elegancy of this approach is founded in its ‘marker-of-self’ nature displaying presumably only minimal antigenicity and immunogenicity. In the future, more studies will have to evaluate which signalling events are triggered by CD47 on artificial structures besides phagocytosis and activation inhibition.
